# Assessment of early wound healing, pain intensity, quality of life and related influencing factors during periodontal surgery: a cross-sectional study

**DOI:** 10.1186/s12903-022-02630-3

**Published:** 2022-12-10

**Authors:** Hongmei Yuan, Qian Liu, Tian Tang, Huan Qin, Lei Zhao, Wen Chen, Shujuan Guo

**Affiliations:** 1 Department of Periodontics, West China Hospital of Stomatology, Sichuan University, Chengdu, 610041 China; 2 State Key Laboratory of Oral Diseases and National Clinical Research Center for Oral Diseases, West China Hospital of Stomatology, Sichuan University, Chengdu, 610041 China

**Keywords:** Wound healing, Pain, Quality of life, Surgical satisfaction, Periodontal surgery

## Abstract

**Background:**

This cross-sectional study assessed early wound healing, pain intensity, quality of life, surgical satisfaction, and related factors during periodontal surgery.

**Methods:**

A total of 369 patients completed the questionnaire before undergoing periodontal surgery (baseline), immediately after the operation (phase I), on the day of suture removal (phase II), and one month later (phase III). The Early Wound Healing Score (EHS) was assessed, and the short-form McGill Pain Questionnaire (SF-MPQ), tooth hypersensitivity visual analog scale (VAS), oral health-related quality of life measure (OHQoL-UK), and surgical satisfaction VAS were administered and analysed.

**Results:**

The EHS was 8.41 ± 2.74 and was influenced by disease severity and surgical factors. Scores on the SF-MPQ, pain intensity scores, and OHQoL-UK scores were significantly increased in phase I and decreased later. Tooth sensitivity decreased significantly one month after periodontal surgery. Psychological factors were positively related to SF-MPQ, pain intensity, OHQoL-UK and tooth sensitivity VAS scores in all phases, while disease severity and surgical factors were only related to these scores at baseline or in phases I/II/III. Surgical acceptance and reoperation willingness continuously decreased after surgery, and all these scores were related to surgical satisfaction.

**Conclusions:**

EHS, pain intensity and quality of life were closely related to disease severity, psychological factors and surgical factors in phase I (i.e., immediately after surgery). The findings suggest that surgical details should be enhanced and that behavioural and psychological interventions measures should be implemented to improve outcomes during periodontal operation and during the early postoperative period as well as to improve patient-oriented periodontal surgery experiences.

*Trial* *registration* This cross-sectional study did not include interventions with human participants, and all the experimental procedures involving humans in this study were approved by the Ethics Committee of West China College of Stomatology, Sichuan University (WCHSIRB-D-2020–284).

**Supplementary Information:**

The online version contains supplementary material available at 10.1186/s12903-022-02630-3.

## Introduction

Periodontal surgery is necessary for individuals with severe periodontitis and mucogingival abnormalities to save affected teeth, improve clinical symptoms, and achieve good occlusal and mastication function or aesthetic requirements [1–5]. Improvements in the clinical outcomes of disease and subjective feelings after periodontal surgery in the long term (3 months or longer) have been widely studied by many researchers in various dimensions [1–4, 6, 7]; however, the short-term effects of periodontal surgery on patient-oriented experiences or early wound healing have rarely been reported [8]. Patient-centred assessments are essential in periodontal treatment, although they are different from the traditional periodontal clinical endpoints. As patient-oriented treatments have gained popularity in periodontal treatment in recent years [9], it has become common to examine the treatment experience of patients after periodontal surgery.

Early wound healing is an essential factor that influences the prognosis of periodontal surgery. Desirable wound closure in the initial two weeks is usually related to lower rates of infection, swelling, pain, and graft loss as well as better quality of life [10–14]. In some types of periodontal surgeries, early wound healing has been reported and described via various methods, but it has not been quantified or standardized [15]. Therefore, it is important to study all types of periodontal surgeries and to assess how the early wound healing score (EHS), which is a quantitative and replicable measure of wound healing [14], and related factors can improve the prognosis of periodontal surgery.

After periodontal surgery, the anaesthetic wears off, and the resulting pain significantly influences the quality of life and surgical experience of individual patients [9, 16]. Therefore, pain management in periodontal surgery is important for clinicians, and the changes in pain intensity and the related factors before and after periodontal surgery are worthy of investigation. As a subjective factor, pain intensity is often studied using visual analog scales (VASs) [17, 18]; furthermore, changes in the sensory and affective dimensions of pain also need to be explored after periodontal surgery. If clinicians know more about the intensity, sensory and affective dimensions of pain after periodontal surgery, they can implement more effective pain management interventions to improve compliance among patients, and patients can also have better experiences with invasive periodontal surgery.

Large-sample studies have revealed that periodontitis significantly decreases oral health-related quality of life [19–23]; however, successful periodontal treatment [5, 24, 25] has been shown to lead to long-term improvements in the psychological and physical aspects of quality of life [32]. Periodontal surgical treatment, as an invasive treatment, has been reported to yield significantly better clinical outcomes [33], but it remains important to examine patient-centred quality of life after periodontal surgery to improve clinical practice and ensure patient compliance, thereby improving clinical endpoints. Thus, it is essential to examine changes in oral health-related quality of life before and after periodontal surgery by performing a comprehensive analysis of related factors.

Therefore, this study evaluated early wound healing, pain intensity, oral health-related quality of life, and surgical satisfaction during periodontal surgery. This study focused on the short-term changes in pain intensity, quality of life outcomes, surgical satisfaction, and related influencing factors, which could help us optimize the details of the surgery and provide references for clinicians to make periodontal surgery more comfortable and beneficial for patients suffering from periodontal disease.

## Materials and methods

This cross-sectional study was performed in accordance with the Declaration of Helsinki and was approved by the Ethics Committee of Institutional Review Board of West China Hospital of Stomatology, Sichuan University (WCHSIRB-D-2020–284). The study was performed in accordance with the STROBE statement. All subjects signed an informed consent form and agreed to participate in this study.

### Subjects enrolled

**Fig. 1:**
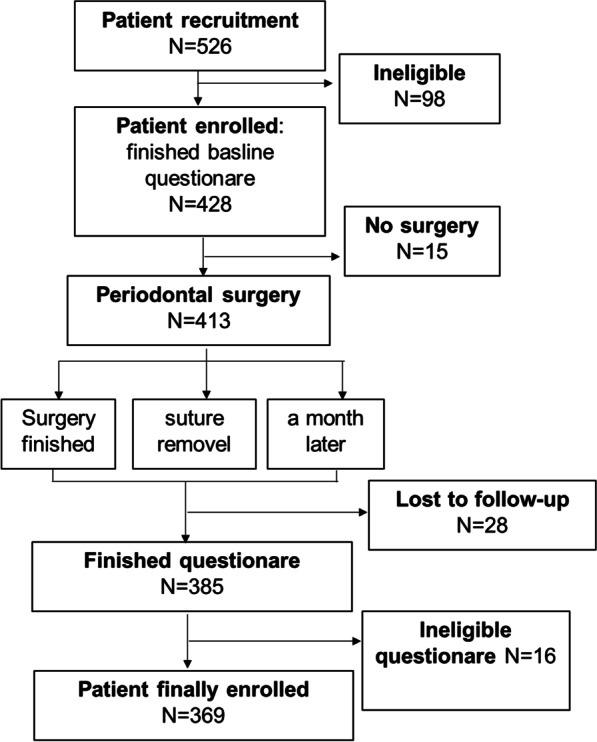
Study design flow chart. A total of 526 individuals were initially recruited, and 98 people were excluded based on the inclusion and exclusion criteria. Fifteen patients did not complete periodontal surgery due to sudden menstruation, the COVID-19 pandemic, time, unnegotiated expenses, or other reasons. A total of 413 subjects finished the periodontal surgery, and 28 people were lost to follow-up. Sixteen patients did not answer important items on the questionnaires (EHS, SF-MPQ, OHQoL-UK). Ultimately, data from 369 eligible subjects were analysed in this study

A total of 369 subjects were recruited from West China Hospital of Stomatology, Sichuan University, between September 2020 and August 2021. The study design is shown in Fig. 1. A total of 526 individuals were initially recruited, and 157 people were excluded for the following reasons: (1) did not meet the inclusion criteria; (2) incomplete periodontal surgery due to any reasons; (3) lost to follow-up; (4) incomplete questionnaires.

The inclusion criteria were as follows: participants who were systematically healthy and had normal oral mucosa; patients who underwent any type of periodontal surgery; patients who were conscious, understood the study procedures, and agreed to participate in this study.

The exclusion criteria were as follows: (1) women who were pregnant or lactating, (2) occurrence of any systemic diseases, (3) oral mucosal disease, tumour, or uncontrolled acute inflammation in the oral cavity, (4) smoking or alcohol abuse, and (5) uncontrolled mental disorders.

### Study procedure

At baseline, the subjects were informed about the study and all questionnaires in a private room for a half hour. They were encouraged to ask questions and told that they could withdraw from the trial for any reason. Once they agreed to participate in this study, they completed a background information questionnaire. This questionnaire was administered before periodontal surgery and was usually completed on the same day as the operation. Furthermore, additional relevant periodontal surgical factors were recorded by nurses during surgery.

Patients completed additional questionnaires at phase I (immediately after surgery), phase II (when sutures were removed), and phase III (a month after periodontal surgery). In phases II and III, wound healing was evaluated by periodontic dentists using the Early Wound Healing Score (EHS). The EHS [14] is composed of 3 dimensions: clinical signs of re-epithelization (CSR), clinical signs of haemostasis (CSH), and clinical signs of inflammation (CSI). The scores of each dimension were summed to yield the EHS. The Kappa value for the EHS was higher than 0.8 (Additional file 1: SI Table I), indicating that the EHS was a reliable measure.

### Questionnaires

The background information questionnaire that was administered at baseline included 9 items assessing demographic information (age, gender, race, residence, profession, education background, population at home, and income), 5 items assessing psychological factors (psychological state, sleep quality, diet, constipation, and stress), 6 items assessing the severity of disease (periodontitis (no, yes), probing depth (≤ 3, 3.1–5.9, ≥ 6), clinical attachment loss (0, ≤ 2, ≥ 3), fracture involvement (no, yes), mobility (no, yes) and gingival recession (no, yes)) and 11 items assessing periodontal surgical factors (surgery type (open flap surgery, guide tissue regeneration, others), number of teeth involved (≤ 3, ≥ 4), surgeon (rich experienced: work more than 10 years, experienced: work more than 5 years and less than 10 years, residents), duration (< 1 h, 1–2 h, > 2 h), complicated operation (no, yes), implanting (no, yes), special equipment (no, yes), suture (no, normal, microsuture), periodontal dressing (no, yes), per-medication (no, yes) and postmedication (no, rinse, rinse + others)).

The short-form McGill pain questionnaire (SF-MPQ)[26], tooth sensitivity visual analog scale (VAS, 10 points) and 16-item United Kingdom Oral Health-related Quality of Life (OHQoL-UK) scale were also administered[27]. The SF-MPQ includes 15 items and two questions. The sensory dimension of pain intensity was assessed by items 1–11, the affective dimension was assessed by items 12–15, and pain intensity was assessed by the two questions (present pain intensity and the VAS). The visual analog scale (VAS) was used to assess tooth sensitivity. The OHQoL-UK scale includes four dimensions: symptoms (comfort, breath odour), physical (eating, appearance, general health, speech, smiling), psychological (relax or sleep, confidence, mood, carefree manner, personality), and social (work, social life, finances, romantic relationships). All descriptors ranged from 1 point (none) to 5 points (very severe). Total scores ranged from 16 (best possible) to 80 (worst possible). The VAS was also used to assess surgical acceptance.

In phases I, II, and III, the SF-MPQ, tooth sensitivity VAS, OHQoL-UK scale, surgical acceptance VAS and reoperation willingness VAS were administered. The EHS was also evaluated in phases II and III.

### Data management and statistical analysis

Paired t tests and one-way ANOVAs were used to compare quantitative data. The chi-square test was used to compare nonparametric data. The Kappa test was performed to analyse the interrater reliability of EHS scores between two evaluators. Multivariate linear logistic regression was used for correlation analysis. *P* < 0.05 was considered significant. All statistical data analyses were performed using SPSS 21.0 and GraphPad 9.3.

## Results

### Overall included subject demographic information

Data from 369 subjects were analysed herein (129 males and 240 females, age 35.58 ± 9.61 years, Table 1). The majority of the subjects were females (65.0%) and undergraduate students (63.1%). Most subjects were of Han ethnicity (91.1%), lived in urban areas (87.8%), had fewer than four people in the household (73.7%), earned less than 120 thousand yuan per year (65.0%), and were unwilling to disclose their profession (51.5%).

**Table 1:** Study group profile

Basic information Mean ± SD/Number(%)	Periodontal condition		Number(%)		
Age	35.58 ± 9.60(369)	PD	≤ 3	113(30.6)	
Gender	male	129(35.0)		3.1–5.9	49(13.3)
	female	240(65.0)		≥ 6	207(56.1)
Race	Han	336(91.1)	CAL	0	64(17.3)
	Other	8(2.2)		≤ 2	70(19.0)
	NR	25(6.8)		≥ 3	235(63.7)
Residence	urban	324(87.8)	FI	No	220(59.6)
	Country	42(11.4)		Yes	149(40.4)
	NR	3(0.8)	Mobility	No	164(44.4)
Profession	employed	134(36.3)		Yes	205(55.6)
	Un-employed	45(12.2)	GR	No	129(35.0)
	NR	190(51.5)		Yes	240(65.0)
Education background	graduate	61(16.5)	Surgery type	flap surgery	133(36.0)
	Undergraduate	233 (63.1)		GTR	131(35.5)
	High school	70 (19.0)		other	105(28.5)
	NR	5 (1.4)	NO. of teeth	≤ 3	222(60.2)
Population at home	≤ 4	272 (73.7)		≥ 4	147(39.8)
	> 4	90(24.4)	Surgeon	Rich Experi	155(42.0)
	NR	7(1.9)		Experi	113(30.6)
In-come (10,000 yuan)	< 5	78 (21.1)		Resident	101(27.4)
	5–12	162 (43.9)	Duration(hour)	< 1	97(26.3)
	12–25	82 (22.2)		1–2	186(50.9)
	≥ 25	31 (8.4)		> 2	84(22.8)
	NR	16 (4.3)	Complicate operate	No	296(80.2)
Psy-state	Not good	65 (17.6)		Yes	73(19.8)
	Good	304 (82.4)	Implanting	No	195(52.8)
Sleep quality	Not good	124 (33.6)		Yes	174(47.2)
	Good	245 (66.4)	Special-equip	No	241(65.3)
Balanced diet	Not good	103(27.9)		Yes	128(34.7)
	Good	266(72.1)	Suture	Not used	12(3.3)
Constipation	Not good	100(27.1)		Normal	161(43.6)
	Good	269(72.9)		Micro	196(53.1)
Work and life Stress	Not good	224(60.7)	PDressing	No	196(53.1)
	Good	145(39.3)		Yes	173(46.9)
Periodontitis	No	136(36.9)	Post-medic	No	8(2.2)
	Yes	233(63.1)		rinse	154(41.7)
Pre-medic	No	304(82.4)		rinse + others	207(56.1)
	Yes	65(17.6)			

*Psy-state* Psychological state, *PD* Probing depth, *CAL* Clinical attachment loss, *GR* Gingival recession, *FI* Furcation involvement, *Special-equip* Special equipment, *PDressing* Periodontal dressing, *Pre/Post-medic* Per-medication/post-medication

Almost two-thirds of the sample reported good outcomes with respect to sleep, diet, and constipation. A total of 82.4% of the sample reported that their psychological state was not good. A total of 60.7% of the sample reported stress in their daily work and life (Table 1).

More than half of the subjects had periodontitis (63.1%), ≥ 6 mm probing depth (56.1%), ≥ 3 mm attachment loss (63.7%), furcation involvement (40.4%), tooth mobility (55.6%) and gingival recession (65.0%).

There was no significant difference among surgery types, implant materials, and periodontal dressings in the enrolled population. Most subjects had fewer than three teeth involved in the operation (60.2%), did not use special equipment (65.3%) or microsutures (53.1%) or preoperative medication (82.4%), and used postoperative medication (97.8%). Most patients underwent operations by highly experienced dentists (42.0%), within 2 h (77.2%), and without complicated operations (80.2%) (Table 1).

### EHS after periodontal surgery and related influencing factors

**Fig. 2:**
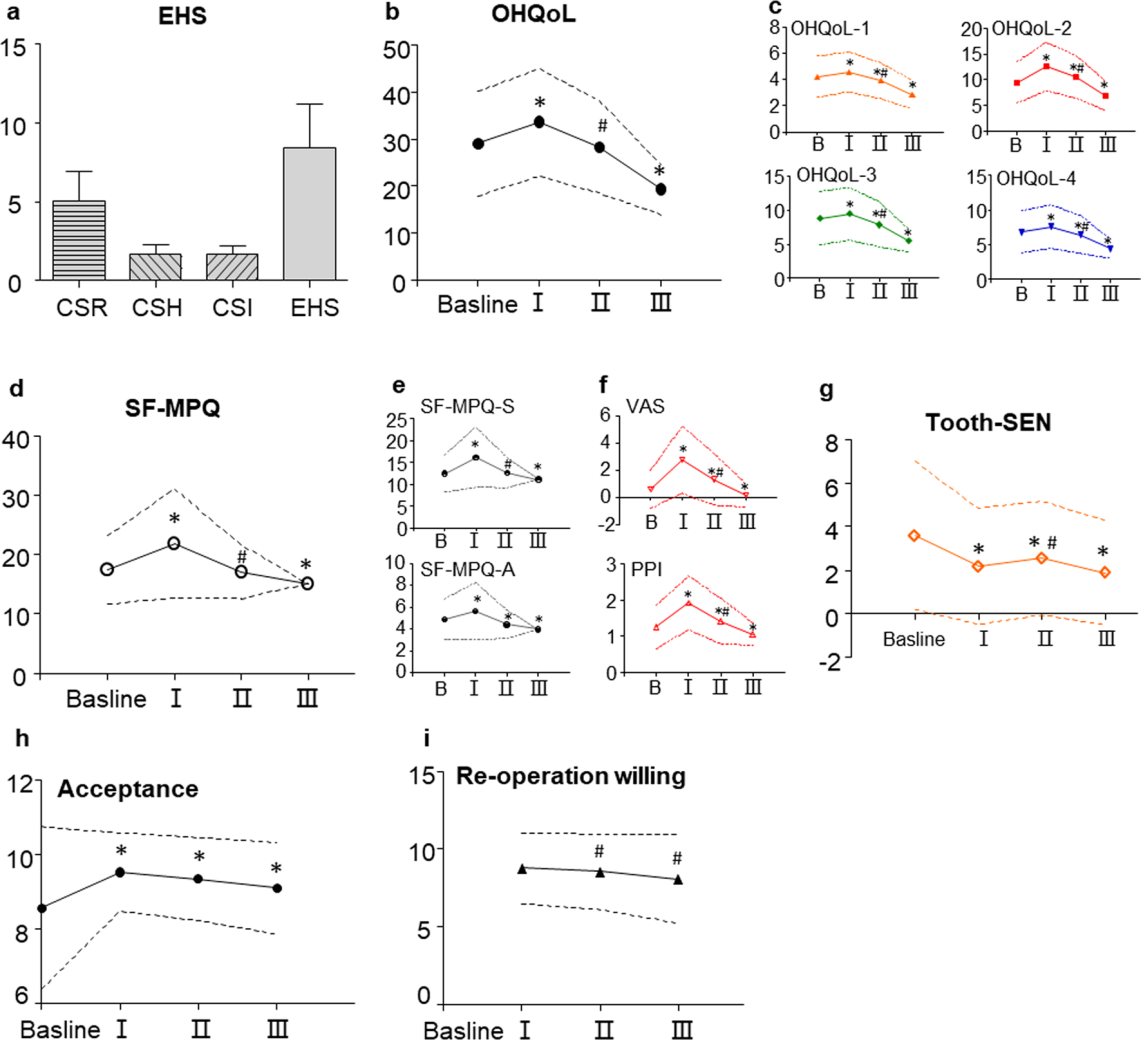
EHS, SF-MPQ, OHQoL-UK, and related parameters during periodontal surgery. **a** CSR (clinical signs of re-epithelization), CSH (clinical signs of haemostasis), CSI (clinical signs of inflammation) and EHS (early wound healing scores, calculated by summing scores on the CSR, CSH, and CSI) when sutures were removed. **b** 16-item United Kingdom Oral Health-related Quality of Life (OHQoL-UK) scores before and after periodontal surgery. **c** OHQoL-UK-1: changes in symptoms (comfort and breath odour). OHQoL-UK-2: changes in physical aspects (eating, appearance, general health, speech, smiling); OHQoL-UK-3: changes in psychological aspects (relax or sleep, confidence, mood, carefree manner, personality); OHQoL-UK-4: changes in social aspects (work, social life, finances, romantic relationships). **d** & **e** &**f**, Changes in SF-MPQ-S (sensory dimension), SF-MPQ-A (affective dimension), and VAS and PPI scores. **G** Changes in the tooth hypersensitivity score. **h**& **i**& **j** Changes in surgical acceptance and reoperation willingness. All data are presented as the mean ± SD, and a paired t test was used, *p < 0.05 compared with baseline. # *p* < 0.05 compared with phase I. B: baseline; I/II/III/IV: phase I/II/III/IV

The EHS (8.41 ± 2.74), CSR score (5.02 ± 1.89), CSH score (1.69 ± 0.61), and CSI score (1.70 ± 0.53) are shown in Fig. 2a. Periodontal dressing, surgeon, and postmedic were related to the EHS (Table 2): Logit(*p*) = − 1.14* Periodontal dressing − 0.6* surgeon + 0.31* postmedic + 10.61. Periodontal dressing, surgeon, gingival recession and postmedic were related to the CSR score: Logit(p) =  − 0.70* Periodontal dressing − 0.43* surgeon + 0.22* postmedic + 6.74. Periodontal dressing, surgeon, and periodontitis were related to the CSH score: Logit(p) =  − 0.27* Periodontal dressing − 0.12* surgeon − 0.16* periodontitis + 2.53. Periodontal dressing and surgeon were related to the CSI score: Logit(p) =  − 0.24* Periodontal dressing − 0.09* surgeon + 2.21. Psychological factors were not related to the EHS (Table 2). The EHS was significantly related to pain intensity (PPI and VAS) and was not related to the sensory or affective dimensions of pain intensity, tooth sensitivity, quality of life, or surgical satisfaction in phase II (Table 50 & Additional file 2: SI Table 2).

**Table 2:** Linear regression analysis of EHS, CSR, CSH and CSI

	B	Beta	95%CI		B	Beta	95%CI
**EHS**				**CSR**			
Constant	10.61		(9.37, 11.85)	Constant	6.74		(5.86, 7.63)
PDressing	− 1.14	− 0.21	(− 1.68, -0.59)	Surgeon	− 0.43	− 0.19	(− 0.67, -0.20)
Surgeon	− 0.6	− 0.18	(− 0.94, − 0.26)	PDressing	− 0.70	− 0.18	(− 1.07,-0.32)
Post-medic	0.31	0.12	(0.04, 0.59)	GR	− 0.46	− 0.12	(− 0.85, -0.08)
**CSH**				Post-medic	0.22	0.12	(0.03, 0.41)
Constant	2.53		(2.21, 2.84)	**CSI**			
PDressing	− 0.27	− 0.22	(− 0.39, − 0.15)	Constant	2.21		(2.03, 2.40)
Surgeon	− 0.12	− 0.16	(− 0.20, -0.04)	PDressing	− 0.24	− 0.23	(− 0.34, − 0.13)
Periodontitis	− 0.16	− 0.13	(− 0.29, − 0.03)	Surgeon	− 0.09	− 0.14	(− 0.15, − 0.03)

The significance of [bold] represents the subcategories within the indexes, which each contain smaller subcategories. Specifically, EHS, CSR, CSH and CSI represent different linear logic analysis results corresponding to EHS, CSR, CSH and CSI

*EHS* Early wound healing score, *CSR* Clinical signs of re-epithelization, *CSH* Clinical signs of haemostasis, *CSI* Clinical signs of inflammation, 95% CI 95% confidence interval

### The pain intensity during periodontal surgery and related factors

The SF-MPQ score significantly increased in phase I, including total scores (21.92 ± 9.19 vs. 17.41 ± 5.78, *p* < 0.05) and scores on the sensory dimension (16.24 ± 6.92 vs. 12.48 ± 4.30, *p* < 0.05), affective dimension (5.68 ± 2.62 vs. 4.93 ± 1.85, *p* < 0.05), intense dimension (PPI, 1.92 ± 0.74 vs. 1.26 ± 0.59, *p* < 0.05 and VAS, 2.78 ± 2.47 vs. 0.61 ± 1.39, *p* < 0.05) and tooth sensitivity (3.60 ± 3.39 vs. 2.18 ± 2.67, *p* < 0.05). When suture removal was performed, the pain intensity for the abovementioned dimensions significantly decreased compared with that in phase I and further decreased with significance in phase III compared with that at baseline (Fig. 2d&e&f&g, Table 3). At baseline, worse psy-state (not good), premedic (yes), and periodontitis (yes) were related to higher SF-MPQ scores (Table 5 & Additional file 2: SI Table 2). In phase I, worse constipation (not good), stress (not good), complicated operation (yes), and premedic (yes) were related to higher SF-MPQ scores. In phase II or III, only psychological factors (worse constipation and Psy-state) contributed to higher SF-MPQ scores.

**Table 3:** SF-MPQ and tooth-sensitivity before and after periodontal surgery

SF-MPQ	baseline	phase I	phase II	phase III
**Total dimension**	**17.41 (5.78)**	21.92 (9.19)*	**17.10 (4.52)#**	15.13 (0.69)*
**Sensory dimension**	**12.48 (4.30)**	16.24 (6.92)*	**12.65 (3.44) #**	11.11 (0.59)*
Throbbing	1.14 **(**0.47**)**	1.57 **(**0.84) *	1.14 **(**0.43**) #**	1.01 **(**0.12) *
Shooting	1.14 **(**0.49**)**	1.62 **(**0.85) *	1.20 **(**0.49**) #**	1.01 **(**0.10) *
Stabbing	1.14 **(**0.49**)**	1.52 **(**0.80) *	1.14 **(**0.43**) #**	1.00 **(**0.05) *
Sharp	1.12 **(**0.49**)**	1.43 **(**0.76) *	1.09 **(**0.36**) #**	1.00 **(**0.05) *
Cramping	1.08 **(**0.40**)**	1.29 **(**0.62) *	1.08 **(**0.31**) #**	1.00 **(**0.00) *
Gnawing	1.10 **(**0.42**)**	1.37 **(**0.71) *	1.12 **(**0.38**) #**	1.01 **(**0.07) *
Hot-burning	1.09 **(**0.39**)**	1.41 **(**0.80) *	1.09 **(**0.33**) #**	1.01 **(**0.10) *
Aching	1.23 **(**0.61**)**	1.51 **(**1.29) *	1.18 **(**0.46**) #**	1.01 **(**0.12) *
Heavy	1.13 **(**0.48**)**	1.42 **(**0.75) *	1.12 **(**0.37**) #**	1.01 **(**0.07) *
Tender	1.20 **(**0.53**)**	1.86 **(**0.87) *	1.38 **(**0.58) *#	1.05 **(**0.27) *
Splitting	1.09 **(**0.42**)**	1.42 **(**0.77) *	1.11 **(**0.35**) #**	1.01 **(**0.12) *
**Affective dimension**	**4.93 (1.85)**	5.68 (2.62)*	4.46 (1.29)*	4.02 (0.23)*
Tiring-exhausting	1.22 **(**0.55**)**	1.52 **(**0.81) *	1.15 **(**0.43**) #**	1.01 **(**0.10) *
Sickening	1.13 **(**0.49**)**	1.40 **(**0.73) *	1.10 **(**0.35**) #**	1.01 **(**0.07) *
Fearful	1.43 **(**0.81**)**	1.51 **(**0.82**)**	1.14 **(**0.42) *#	1.01 **(**0.07) *
Punishing-cruel	1.16 **(**0.51**)**	1.29 **(**0.67) *	1.08 **(**0.34) * #	1.00 **(**0.05) *
**Intense dimension**				
PPI	1.26 (0.59)	1.92 (0.74) *	1.42 (0.63) * #	1.06 (0.31) *
VAS	0.61 (1.39)	2.78 (2.47) *	1.33 (1.89) * #	0.16 (0.85) *
**Tooth-SEN**	**3.60 (3.39)**	2.18 (2.67) *	2.56 (2.61) * #	1.89 (2.40) *

The significance of [bold] represents sensory dimension is the sum of the following 11 items: Throbbing, Shooting, Stabbing, Sharp, Cramping, Gnawing, Hot-burning, Aching, Heavy, Tender, Splitting. Affective dimension is the sum of the following 4 items: Tiring-exhausting, Sickening, Fearful, Punishing-cruel. Total dimension is the sum of the above 15 items. The intense dimension contains PPI and VAS of pain. Tooth-SEN is a subgroup independent of all of the above indicators used to assess dental sensitivity

All data were present with Mean (SD) and Paired t test were used, *p < 0.05 compared with baseline, # *p* < 0.05 phase II compared with phase I, *SF-MPQ* Short-form McGill pain questionnaire, *PPI* The present pain intensity, *VAS* The visual analogue of pain, *Tooth-SEN* Tooth hypersensitivity

### Life quality changes during periodontal surgery and related factors

The OHQoL-UK scores, including total scores (29.11 ± 11.19 vs. 33.63 ± 11.41, *p* < 0.05), scores on the symptoms dimension (4.24 ± 1.57 vs. 4.60 ± 1.53, *p* < 0.05), scores on the physical dimension (9.47 ± 3.99 vs. 12.58 ± 4.67, *p* < 0.05), scores on the psychological dimension (8.80 ± 3.92 vs. 9.43 ± 3.85, *p* < 0.05), and scores on the social dimension (6.79 ± 3.05 vs. 7.60 ± 3.14, *p* < 0.05) were significantly increased after periodontal surgery in phase I and significantly decreased in phase II or III compared with those in phase I or at baseline (Table 4, & Fig. 2b&c). At baseline, sleep (not good), stress (not good), constipation (not good), tooth mobility (yes), and higher SF-MPQ scores were significantly related to high OHQoL-UK scores. After periodontal surgery, psychological factors (sleep, stress), disease severity (FI, GR, periodontitis), periodontal surgical factors (fewer teeth involved in the operation, postmedic) and pain intensity (SF-MPQ and Tooth-SEN) contributed to worse OHQoL-UK scores.

**Table 4:** 16-item United Kingdom oral health-related quality of life (OHQoL-UK) scores before and after periodontal surgery

OHQoL-UK	baseline	phase I	phase II	phase III
**Total aspects**	**29.11(11.19)**	**33.63 (11.41)** *	**28.35(9.83)** #	**19.34(5.35)** *
**Symptoms aspects**	**4.24 (1.57)**	**4.60 (1.53)** *	**3.92 (1.37)** *#	**2.87 (1.11)** *
Comfort	2.32 (0.92)	2.84 (1.08)*	2.34 (0.88)#	1.73 (0.91)*
Breath odour	1.92 (0.88)	1.77 (0.81) *	1.57 (0.76) *#	1.13 (0.42) *
**Physical aspects**	**9.47 (3.99)**	**12.58 (4.67)** *	**10.57 (4.11)** *#	**6.89 (2.87)** *
Eating	2.01 (1.05)	2.95 (1.14) *	2.69 (1.02) *#	1.87 (0.94) *
Appearance	2.16 (1.16)	2.28 (1.02)	1.99 (1.01) *#	1.30 (0.75) *
General health	1.78 (0.86)	1.84 (0.79)	1.58 (0.68) *#	1.06 (0.26) *
Speech	1.69 (0.84)	2.40 (1.13) *	1.87 (0.93) *#	1.17 (0.56) *
Smiling	1.85 (1.02)	2.64 (1.18) *	2.03 (1.04) *#	1.24 (0.70) *
**Psychological aspects**	**8.80 (3.92)**	**9.43 (3.85)** *	**7.89(3.30)** *#	**5.48 (1.66)** *
Relax or sleep	1.64 (0.78)	2.13 (0.98) *	1.62 (0.76) #	1.07 (0.30) *
Confidence	1.81 (0.93)	1.82 (0.83)	1.58 (0.71) *#	1.13 (0.49) *
Mood	1.78 (0.91)	1.87 (0.84)	1.59 (0.72) *#	1.14 (0.51) *
Carefree manner	1.90 (0.94)	1.86 (0.86)	1.61 (0.76) *#	1.09 (0.39) *
Personality	1.66 (0.79)	1.76 (0.77) *	1.52 (0.68) *#	1.06 (0.25) *#
**Social aspects**	**6.79 (3.05)**	**7.60 (3.14)** *	**6.42 (2.73)** *#	**4.39 (1.42)** *
Work	1.68 (0.81)	2.00 (0.98) *	1.62 (0.78) #	1.11 (0.45) *
Social life	1.73 (0.88)	2.04 (0.98) *	1.69 (0.85) #	1.12 (0.47) *
Finances	1.80 (0.88)	1.90 (0.85) *	1.64 (0.79) *#	1.11 (0.43) *
Romantic relationships	1.60 (0.77)	1.69 (0.73) *	1.49 (0.63) *#	1.05 (0.25) *

The significance of [bold] represents symptoms aspects is the sum of the following 2 items: Comfort and Breath odour. Physical aspects is the sum of the following 5 items: Eating, Appearance, General health, Speech, Smiling. Psychological aspects is the sum of the following 5 items: Relax or sleep, Confidence, Mood, Carefree manner. Personality. Social aspects is the sum of the following 4 items: Work, Social life, Finances, Romantic relationships. Total aspects is the sum of the above 16 items

All data were present with Mean (SD) and Paired t test were used, *p < 0.05 compared with baseline, # *p* < 0.05 phase II compared with phase I, OHQoL-UK: 16-item United Kingdom oral health related quality-of-life measure

### Surgical satisfaction during periodontal surgery and related factors

Surgical acceptance scores increased in phase I (8.56 ± 2.18 vs. 9.52 ± 1.05, *p* < 0.05) and decreased continuously in phases II (9.34 ± 1.11) and III (9.09 ± 1.23), but they remained significantly higher than the baseline scores (Fig. 2h). The reoperation willingness continuously decreased in phases II (8.53 ± 2.43) and III (8.05 ± 2.84) compared with that in phase I (8.75 ± 2.30) (Fig. 2i). At baseline, the OHQoL-UK score was related to surgical acceptance. After periodontal surgery, psychological factors (psy state, balanced diet, and sleep), tooth mobility, periodontal surgical factors (duration and special equipment), pain intensity (SF-MPQ, PPI, and Tooth-SEN), and OHQoL-UK scores influenced surgical acceptance (Additional file 2: SI Table 2). After surgery, better surgical acceptance, less pain intensity (PPI), worse disease severity (CAL, FI), stress, and nonimplanting were related to lower reoperation willingness (Table 5).

**Table 5:** Linear regression analysis of the SF-MPQ, OHQoL-UK and re-operation willing

	B	Beta	95%CI		B	Beta	95%CI
**SF-MPQ**				**OHQoL-UK**			
**Constant (b)**	22.90		(19.62, 26.18)	**Constant (b)**	35.89		(29.93, 41.85)
Psy-state	− 1.96	− 0.13	(− 3.50, − 0.42)	SF-MPQ	0.63	0.33	(0.46, 0.80)
Pre-medic	2.55	0.17	(0.98, 4.13)	WL Stress	− 4.97	− 0.22	(− 7.06, -2.89)
Periodontitis	− 1.73	− 0.14	(− 2.98, -0.48)	Sleep quality	− 4.44	− 0.19	(− 6.77, -2.12)
**Constant (I)**	29.75			Mobility	2.24	0.10	(0.27, 4.21)
Constipation	− 3.64	− 0.18	(25.53, 33.97)	Constipation	− 2.58	− 0.10	(− 5.06, − 0.09)
Complic-ope	3.64	0.16	(− 5.78, − 1.50)	**Constant (I)**	29.45		(24.23, 34.67)
Pre-medic	2.95	0.12	(1.34, 5.94)	SF-MPQ	0.62	0.50	(0.50, 0.75)
WL Stress	− 2.01	− 0.11	(0.56, 5.35)	Sleep quality	− 4.23	− 0.18	(− 6.22, − 2.24)
**Constant (II)**	22.86		(− 3.96,-0.07)	Periodontitis	− 2.81	− 0.12	(− 4.73, − 0.89)
Psy-state	− 1.90	− 0.16		Tooth-SEN	0.49	0.11	(0.08, 0.90)
Constipation	− 1.33	− 0.13	(20.44, 25.28)	**Constant (II)**	18.69		(12.58, 24.79)
**Constant (III)**	15.49		(− 3.19, − 0.60)	SF-MPQ	0.73	0.34	(0.52, 0.93)
Psy-state	− 0.20	− 0.11	(− 2.44, − 0.23)	Tooth-SEN	0.62	0.17	(0.27, 0.97)
**Re-operation willing**		Sleep quality	− 2.44	− 0.12	(− 4.39, -0.48)		
**Constant (I)**	0.11		(-1.98, 2.19)	WL Stress	− 2.63	− 0.13	(− 4.49, -0.78)
Surgi-accept	0.90	0.46	(0.71, 1.08)	NO. of tooth	2.37	0.12	(0.56, 4.18)
CAL	0.44	0.14	(0.16, 0.73)	**Constant (III)**	− 2.80		(− 14.45, 8.84)
PPI	− 0.44	− 0.14	(− 0.75, − 0.13)	SF-MPQ	1.88	0.24	(1.13, 2.62)
**Constant (II)**	− 1.08		(− 2.92, 0.76)	Sleep quality	− 2.10	− 0.19	(− 3.14, − 1.06)
Surgi-accept	1.13	0.52	(0.94, 1.33)	Tooth-SEN	0.39	0.17	(0.16, 0.61)
WL Stress	− 0.63	− 0.15	(− 1.08, − 0.19)	Post-medic	0.71	0.13	(0.20, 1.22)
**Constant (III)**	− 0.62		(− 2.30, 1.06)	Periodontitis	− 2.45	− 0.22	(− 3.68, − 1.22)
Surgi-accept	0.98	0.49	(0.80, 1.16)	FI	− 1.49	− 0.14	(− 2.71, − 0.28)
Implanting	− 0.68	− 0.12	(− 1.20, -0.17)	GR	− 1.26	− 0.11	(− 2.36, − 0.15)
FI	0.60	0.10	(0.08, 1.13)				

*Complic-ope* Complicate operate, *WL Stress* Work and life stress, *Surgi-accept* Surgical acceptance

## Discussion

Patient-centred assessments are essential in periodontal treatment, and their focus may be different from that of traditional clinical endpoints; thus, they have been ignored when many researchers have widely reported improved clinical outcomes after periodontal surgery in various dimensions [1–4, 6–8]. Here, we studied and depicted patient-centred assessments and found some potential details related to better surgical effects and patient experience during periodontal surgery.

Wound closure has been universally considered a crucial part of periodontal surgical treatment [10, 11] and is related to higher pain intensity and lower quality of life. The initial few weeks after periodontal surgery are critical for wound healing stability [12, 13], so we evaluated the early wound healing score [14] and some related factors influencing wound healing, which would also potentially influence patient-oriented assessments, such as pain intensity and quality of life. The EHS at post-surgery suture removal was 8.41 ± 2.74 in this study, which was similar to the score reported by Rojas (8.10 ± 1.00) after papillary preservation flaps with bone and bovine pericardial membrane grafts in periodontitis patients [28]. Lavu et al. reported that an EHS of 8.14 ± 1.41 on the 10th day after the laterally closed tunnel technique for the management of gingival recession [29]. The high standard deviation of the EHS in this study may be due to the suture removal time ranging from 1 to 2 weeks according to various types of surgery, including GTR, GBR, mucogingival surgery, gingival resection, crown lengthening, and implant surgery. This study indicated that a surgeon with rich experience was positively related to better EHS. The usage of periodontal dressing was negatively related to early wound healing. This might be because periodontal dressings are usually applied after complicated surgery with difficulty in wound closure. Postmedics would benefit wound healing, and worse periodontal status (gingiva recession or periodontitis) is related to poor wound healing. The wound healing score was significantly related to the pain intensity (PPI, VAS) and did not influence the sensory or affective dimensions of pain or tooth sensitivity in phase II. Additionally, EHS was not related to quality of life or surgical satisfaction after periodontal surgery, and this might be because other synergistic influencing factors, such as pain intensity, were added to the logistic analysis of quality of life and surgical satisfaction.

Pain intensity after periodontal surgery is usually assessing using the VAS. However, the VAS only represents the intensity of pain, not the sensory and affective aspects of pain intensity [26]. In this study, the short-form McGill Pain Questionnaire (SF-MPQ) assessed the sensory, affective, and intensity dimensions of pain intensity, and the VAS was also used to assess tooth sensitivity [26]. In this study, tooth sensitivity was significantly decreased from baseline to phase III. We should interpret result with caution due to the various types of periodontal surgeries. Patients with gingival recession who need root coverage surgery usually report tooth sensitivity before surgery; on the other hand, patients with periodontitis undergoing flap surgery usually report tooth sensitivity after surgery. After periodontal surgery, worse psychological outcomes (stress and constipation) were significantly related to tooth sensitivity. Thus, improving psychological states would help relieve tooth sensitivity. All SF-MPQ scores were significantly lower in phases II-III than at baseline or phase I. The results revealed that periodontal surgery was beneficial for decreasing pain levels at one month[31]. All dimensions of pain intensity increased in phase I, especially immediately after surgery. In this phase, periodontal surgery factors (complicated operation, premedic) and worse psychological outcomes (constipation and stress) were related to higher levels of pain intensity. Therefore, simplifying the complicated operations during surgery may lead to lower levels of pain. The use of premedics was related to a high SF-MPQ score, which might be because patients with more complicated disease and operations or patients who were sensitive to pain were usually prescribed painkillers or antibiotics; therefore, higher pain levels were reported by them at baseline. Patients with worse psychological outcomes reported higher pain scores at baseline as well as in phases I, II and III (Table 5). Therefore, it is important for clinicians to put forth efforts to improve patients' mental state through various methods during periodontal treatment, thus enhancing patient-centred treatment.

Oral health-related quality of life has been widely examined among periodontitis patients [19–23] during nonsurgical [6, 24, 32] and surgical treatment [5, 7, 25, 33]. Successful periodontal therapies (both nonsurgical and surgical treatment) have been shown to have a positive impact on OHrQoL both in the short term [24] and the long term [32]. Periodontal surgical treatment has been shown to significantly improve OHRQoL as well as various clinical parameters[33]. In this study, periodontal surgery significantly improved quality of life at one month (29.11 ± 11.19 vs. 19.34 ± 5.35, *p* < 0.05). Quality of life in phase I decreased compared with that at baseline, which directly affected the reoperation willingness after periodontal surgery. Therefore, understanding the factors that influence OHRQoL among periodontal surgery patients is important for clinicians. We found that patients with more severe pain, tooth mobility, and periodontitis reported worse OHRQoL at baseline and in phase I, which was consistent with previous studies reporting that periodontitis was related to worse OHRQoL [19–23]. Poor sleep, stress, and constipation were related to worse OHRQoL. Goh’s study focusing on psychological factors also found that combinations of depression, anxiety and stress led to worse OHRQoL in patients with periodontitis [34]. In phases II and III, pain intensity (SF-MPQ and Tooth-SEN), psychological factors (sleep, stress), severity of disease (FI, GR, periodontitis), and periodontal surgical factors (fewer teeth involved in the operation, postmedic) also contributed to the changes in the OHQoL-UK score. Rawlinson et al. found that psychological factors significantly influenced quality of life during surgery [35], which was also observed in our study. Here, we found that severe stress and sleep quality were related to worse quality of life before and after periodontal surgery. Therefore, improving patients' psychological state during periodontal treatment would lead to multiple benefits, such as decreasing pain intensity and increasing quality of life, thereby yielding favourable patient-centred treatment outcomes in clinical practice. The VAS scores for surgical acceptance and reoperation willingness were also reported. Surgical acceptance increased immediately after surgery. Both surgical acceptance and reoperation willingness decreased little one month after surgery. Although these indicators decreased with significance, the absolute value changed slightly after surgery, and the surgery acceptance was still higher than that at baseline. Therefore, improving surgery satisfaction is necessary by altering surgery factors, psychology factors, quality of life and pain intensity.

This study also had limitations. First, the patients were mainly of Han ethnicity and lived in southwestern China. However, there were numerous exclusion criteria, such as smoking, alcohol abuse, pregnancy, systemic diseases, and other oral diseases, all of which would have significantly influenced the main results (wound healing, pain intensity, quality of life). Second, certain parameters, such as quality of life and pain intensity, have inherent bias because they are self-reported by individual patients. However, the use of reliable questionnaires to assess the same parameters minimized the risk of bias in this study.

## Conclusion

Herein, early wound healing after periodontal surgery was generally satisfactory when sutures were removed, and the EHS was related to the severity of periodontal diseases and surgical factors. Pain intensity and quality of life decreased immediately after periodontal surgery and returned to baseline levels in one month; these parameters were influenced by disease severity, surgical factors and psychological factors, thus providing guidance on which factors should be optimized after periodontal surgery. In conclusion, this study revealed changes in patient-centred assessments after periodontal surgery and provided us with potential methods for improving patient experiences with periodontal surgery, such as optimizing surgery details and implementing behavioural and psychological interventions.

## Supplementary Information

**Additional file 1**.** SI Table 1**. The Kappa test for EHS score.

**Additional file 1**.** SI Table 2**. Linear regression model of PPI, VAS of pain intense, sensory dimension of SF-MPQ, Tooth-SEN and surgical acceptance.

## Data Availability

The data of the findings in this study are available from the corresponding author upon reasonable request.
