# Supplementary material for: Assessment of early wound healing, pain intensity, quality of life and related influencing factors during periodontal surgery: a cross-sectional study
Source: BMC Oral Health. 2022 Dec 10;22:596. doi: 10.1186/s12903-022-02630-3 (PMC9741525; doi:10.1186/s12903-022-02630-3)
Supplement: Supplementary file 1 — Additional file 1. SI Table 1. The Kappa test for EHS score. [file 12903_2022_2630_MOESM1_ESM.docx]

**SI Table 1. The Kappa test for EHS score**

|  | value | Asymp. Std. Error^a^ | Approx. T^b^ | Approx. Sig. |
| --- | --- | --- | --- | --- |
| Measure of agreement kappa | **0.816** | 0.101 | 4.907 | 0.000 |
| N of Valid Cases | 36 |  |  |  |

a. Not assuming the unll hypothesis.

b. Using the asymptotic standard error assuming the null hypothesis.

**Tables legends**

**SI Table 1.** The Kappa test for EHS score. The EHS scores were given by 2 different professors in periodontics. A Kappa test on EHS scores was done before study and the kappa values (kappa values=0.82) were higher than 0.8, thus ensuring the EHS scores between the two dentists were reproducible.
