# Supplementary material for: Assessment of early wound healing, pain intensity, quality of life and related influencing factors during periodontal surgery: a cross-sectional study
Source: BMC Oral Health. 2022 Dec 10;22:596. doi: 10.1186/s12903-022-02630-3 (PMC9741525; doi:10.1186/s12903-022-02630-3)
Supplement: Supplementary file 2 — Additional file 1. SI Table 2. Linear regression model of PPI, VAS of pain intense, sensory dimension of SF-MPQ, Tooth-SEN and surgical acceptance. [file 12903_2022_2630_MOESM2_ESM.docx]

**SI Table 2. Linear regression model of PPI, VAS of pain intense, sensory dimension of SF-MPQ, Tooth-SEN and surgical acceptance**

|  | **B** | **Beta** | **T** | **95%CI** |  | **B** | **Beta** | **T** | **95%CI** |
| --- | --- | --- | --- | --- | --- | --- | --- | --- | --- |
| **PPI** |  |  |  |  | **Tooth-SEN** |  |  |  |  |
| **Constant (b)** | 1.20 |  | 30.41(0.00) | (1.12, 1.27) | **Constant (b)** | 2.00 |  | 3.94(0.00) | (1.00, 3.00) |
| FI | 0.16 | 0.13 | 2.59(0.01) | (0.04, 0.28) | Mobility | 1.32 | 0.19 | 3.77(0.00) | (0.63, 2.01) |
| **Constant (I)** | 2.24 |  | 11.06(0.00) | (1.84, 2.63) | Surgery type | 0.48 | 0.11 | 2.19(0.03) | (0.05, 0.91) |
| Special-equip | 0.24 | 0.15 | 2.98(0.00) | (0.08, 0.39) | **Constant (I)** | 3.02 |  | 7.08(0.00) | (2.18, 3.86) |
| FI | -0.21 | -0.14 | -2.66(0.01) | (-0.36, -0.05) | WL stress | -0.80 | -0.15 | -2.87(0.00) | (-1.35, -0.25) |
| Constipation | -0.20 | -0.12 | -2.37(0.02) | (-0.36, -0.03) | Special-equip | 0.78 | 0.14 | 2.74(0.01) | (0.22, 1.35) |
| Duration | 0.15 | 0.14 | 2.77(0.01) | (0.04, 0.26) | **Constant (II)** | 3.63 |  | 3.98(0.00) | (1.84, 5.43) |
| PDressing | -0.19 | -0.13 | -2.44(0.02) | (-0.34, -0.04) | Periodontitis | -0.75 | -0.14 | -2.68(0.01) | (-1.30, -0.20) |
| **Constant (II)** | 2.11 |  | 11.04(0.00) | (1.73. 2.49) | Suture | -0.59 | -0.13 | -2.47(0.01) | (-1.06, -0.12) |
| Psy-state | -0.26 | -0.16 | -3.08(0.00) | (-0.43, -0.10) | PDressing | 0.73 | 0.14 | 2.73(0.01) | (0.20, 1.26) |
| EHS | -0.02 | -0.11 | -2.10(0.04) | (-0.05, -0.00) | NO. of tooth | 0.61 | 0.11 | 2.26(0.02) | (0.08, 1.13) |
| **Constant (III)** | 1.21 |  | 15.21(0.00) | (1.05, 1.36) | Constipation | -0.63 | -0.11 | -2.11(0.04) | (-1.21, -0.04) |
| Psy-state | -0.20 | -0.25 | -4.06 (0.00) | (-0.30, -0.10) | **Constant (III)** | 3.22 |  | 4.02(0.00) | (1.65, 4.80) |
| Sleep | 0.13 | 0.20 | 3.35(0.00) | (0.06, 0.21) | Periodontitis | -1.12 | -0.23 | -4.50(0.00) | (-1.61, -0.63) |
| **VAS of pain intense** | |  |  |  | PDressing | 0.62 | 0.13 | 2.58(0.01) | (-0.15, 1.09) |
| **Constant (b)** | 1.33 |  | 4.52(0.00) | (0.75, 1.91) | Constipation | -0.97 | -0.18 | -3.41(0.00) | (-1.54, -0.41) |
| Constipation | -0.40 | -0.13 | -2.44 (0.02) | (-0.73, -0.08) | Psy-state | 1.00 | 0.16 | 3.01(0.00) | (0.35, 1.66) |
| **Constant (I)** | 3.88 |  | 7.30(0.00) | (2.84, 4.93) | Special-equip | -0.56 | -0.11 | -2.25(0.03) | (-1.04, -0.07) |
| Constipation | -0.86 | -0.15 | -3.09(0.00) | (-1.40, -0.31) | Suture | -0.44 | -0.10 | -2.07(0.04) | (-0.86, -0.02) |
| FI | -0.98 | -0.20 | -3.77(0.00) | (-1.49, -0.47) | **Sugerical Acceptance** | |  |  |  |
| Mobility | 0.95 | 0.19 | 3.69(0.00) | (0.44, 1.45) | **Constant (b)** | 9.66 |  | 30.22(0.00) | (9.03, 10.28) |
| Special-equip | 0.72 | 0.14 | 2.80(0.01) | (0.21, 1.23) | OHQoL-UK | -0.04 | -0.19 | -3.68(0.00) | (-0.06, -0.02) |
| **Constant (II)** | 3.28 |  | 6.67(0.00) | (2.31, 4.25) | **Constant (I)** | 9.99 |  | 24.96(0.00) | (9.20, 10.78) |
| Balanced diet | -0.79 | -0.19 | -3.66(0.00) | (-1.21, -0.36) | OHQoL-UK | -0.02 | -0.17 | -2.60(0.01) | (-0.03, 0.00) |
| EHS | -0.07 | -0.10 | -2.02(0.04) | (-0.14, 0.00) | Psy-state | 0.57 | 0.18 | 3.56(0.00) | (0.25, 0.88) |
| **Constant (III)** | 1.77 |  | 5.63(0.00) | (1.15, 2.39) | Duration | -0.28 | -0.16 | -3.24(0.00) | (-0.46,-0.11) |
| Psy-state | -0.31 | -0.14 | -2.76(0.01) | (-0.53, -0.09) | SF-MPQ | -0.02 | -0.18 | -2.88(0.00) | (-0.04, -0.0) |
| NO. of tooth | -0.28 | -0.16 | -3.06(0.00) | (-0.46, -0.10) | **Constant (II)** | 9.92 |  | 25.99(0.00) | (9.17, 10.67) |
| surgeon | -0.16 | -0.16 | -2.91(0.00) | (-0.27, -0.05) | OHQoL-UK | -0.03 | -0.26 | -5.30(0.00) | (-0.04, -0.02) |
| Periodontitis | -0.26 | -0.15 | -2.75(0.01) | (-0.44, -0.07) | Balanced diet | 0.63 | 0.25 | 3.99(0.00) | (0.32, 0.93) |
| **Sensory dimension of SF-MPQ** | | |  |  | PPI | -0.34 | -0.19 | -3.90(0.00) | (-0.51, -0.17) |
| **Constant (b)** | 16.045 |  | 13.006(0.00) | (13.62, 18.47) | Duration | -0.22 | -0.14 | -2.89(0.00) | (-0.37, -0.07) |
| Pre-medic | 1.901 | .169 | 3.194(0.00) | (0.73, 3.07) | Special-equip | -0.25 | -0.11 | -2.30(0.02) | (-0.47, -0.04) |
| Periodontitis | -1.278 | -.144 | -2.714(0.01) | (-2.20, -0.35) | Sleep | -0.39 | -0.16 | -2.63(0.01) | (-0.67, -0.10) |
| Psy-state | -1.182 | -.104 | -2.039(0.04) | (-2.32, -0.42) | Psy-state | 0.42 | 0.14 | 2.37(0.02) | (0.07, 0.78) |
| **Constant (I)** | 20.196 |  | 13.845(0.00) | (17.33, 23.07) | **Constant (III)** | 4.43 |  | 2.47(0.01) | (0.90, 7.96) |
| Constipation | -2.867 | -.186 | -3.625(0.00) | (-4.42, -1.31) | OHQoL-UK | -0.07 | -0.25 | -4.78(0.00) | (-0.09, -0.04) |
| Complic-ope | 2.658 | .154 | 3.032(0.00) | (0.93, 4.38) | Balanced diet | 0.57 | 0.18 | 3.71(0.00) | (0.27, 0.87) |
| Pre-medic | 2.544 | .141 | 2.761(0.01) | (0.73, 4.36) | Tooth-SEN | -0.09 | -0.15 | -2.86(0.00) | (-0.14, -0.03) |
| **Constant (II)** | 16.837 |  | 17.908(0.00) | (14.99, 18.69) | Mobility | -0.32 | -0.11 | -2.34(0.02) | (-0.59, -0.05) |
| Psy-state | -1.416 | -.156 | -2.815(0.01) | (-2.41, -0.43) | SF-MPQ | 0.39 | 0.19 | 3.03(0.00) | (0.14, 0.64) |
| Constipation | -.930 | -.120 | -2.168(0.03) | (-1.78, -0.09) | PPI | -0.67 | -0.15 | -2.43(0.02) | (-1.22, -0.13) |
| **Constant (III)** | 11.275 |  | 129.534(0.00) | (11.10, 11.45) |  |  |  |  |  |
| Suture | -.111 | -.106 | -2.047(0.04) | (-0.22, 0.00) |  |  |  |  |  |

Psy-state: psychological state; PD: probing depth; CAL: clinical attachment loss; GR: gingival recession; FI: furcation involvement; Special-equip: special equipment; PDressing: periodontal dressing; Pre/Post-medic: permedication/postmedication; EHS early wound healing scores, CSR: clinical signs of re-epithelization, CSH: clinical signs of haemostasis, CSI: clinical signs of inﬂammation, CI: confidence interval. Complic-ope: complicate operate; WL Stress: work and life stress; Surgi-accept: surgical acceptance; PPI: the present pain intensity; VAS: the visual analogue of pain. Tooth-SEN: Tooth hypersensitivity. OHQoL-UK: 16-item United Kingdom oral health related quality-of-life measure.

**Tables legends**

**SI Table 2.** Linear regression model of PPI, VAS of pain intense, sensory dimension of SF-MPQ, Tooth-SEN and surgical acceptance. Multiple liner logistic regression presenting the influence of continuous variables on the PPI, VAS of pain intense, sensory dimension of SF-MPQ, Tooth-SEN and surgical acceptance with regression coefficients (B) regression coefficients (Beta), t (p value) and correspondent 95% confidence intervals (95% CI).
